# Association Between Gestational Diabetes Mellitus and the Risks of Type-Specific Cardiovascular Diseases

**DOI:** 10.3389/fpubh.2022.940335

**Published:** 2022-07-05

**Authors:** Yuanyuan Mao, Wenbin Hu, Bin Xia, Li Liu, Xia Han, Qin Liu

**Affiliations:** ^1^Department of Obstetrics and Gynecology, Suzhou Medical College of Soochow University, Suzhou, China; ^2^Department of Obstetrics and Gynecology, The First People's Hospital of Kunshan Affiliated With Jiangsu University, Suzhou, China; ^3^Department of Chronic and Noncommunicable Disease Control and Preventions, The Kunshan Center for Disease Control and Prevention, Suzhou, China; ^4^Department of Administration, Maternal and Child Health Institution, Kunshan, China

**Keywords:** gestational diabetes mellitus, type 2 diabetes, coronary heart disease, heart failure, cerebrovascular disease

## Abstract

**Objective:**

Gestational diabetes mellitus (GDM) has been linked to subsequent overall cardiovascular diseases. However, evidence on the associations of GDM with type-specific cardiovascular diseases is lacking, and findings on the potential impact of type 2 diabetes on the associations are not consistent. This study aimed to explore the associations between GDM and the risks of type-specific cardiovascular diseases.

**Methods:**

Data were from 12,025 women (≥20 years) who had delivered at least one live birth in the National Health and Nutrition Examination Survey, 2007–2018. GDM history and type-specific cardiovascular diseases including coronary heart disease (CHD), heart failure and stroke were defined by self-report. We also combined our results with those from previously related publications on the associations between GDM and risks of type-specific cardiovascular diseases with a random-effect model.

**Results:**

Compared with women without GDM, the multivariable-adjusted odds ratios (95% confidence intervals) were 1.82 (1.21–2.72) for CHD, 1.43 (0.80–2.53) for heart failure, and 1.19 (0.76–1.86) for stroke among women with a history of GDM. Type 2 diabetes was associated with 43.90, 67.44, and 63.16% of the excess odds of CHD, heart failure and stroke associated with GDM, respectively. Combining results from this study with those from previously related studies yielded odds ratios (95% confidence intervals) of 1.81 (1.60–2.05) for CHD (12 studies, 7,615,322 participants, *I*^2^= 72.6%), 1.66 (1.25–2.21) for heart failure (5 studies, 4,491,665 participants, *I*^2^= 88.6%), and 1.25 (1.07–1.46) for cerebrovascular disease (9 studies, 6,090,848 participants, *I*^2^= 77.8%).

**Conclusions:**

GDM showed stronger associations with coronary heart diseases and heart failure than cerebrovascular disease, and the excess risks are attributable, in part, to type 2 diabetes.

## Introduction

Globally, the prevalence of gestational diabetes mellitus (GDM) was estimated at 14.0%, and the standardized prevalence of GDM in low-, middle- and high-income countries was 12.7, 9.2, and 14.2% (1), respectively. GDM could increase the risk of long-term complications including type 2 diabetes, metabolic syndrome and hypertension (2–6), which are detrimental to cardiovascular health. In particular, women with a history of GDM have a nearly 10-fold higher risk of developing type 2 diabetes than women without a history of GDM (2). A recent meta-analysis of 9 studies found a 2-fold higher risk of subsequent overall cardiovascular diseases in women with a history of GDM than women without GDM (7). Therefore, the diagnosis of GDM provides unique opportunities for early intervention and risk modification of cardiovascular diseases (8, 9), although screening for cardiovascular disease has not been included in current guidelines for the care of women with a history of GDM (10, 11).

However, evidence on the associations between GDM and type-specific cardiovascular diseases is lacking (7, 12), and findings from a few recent studies on the associations between GDM and type-specific cardiovascular diseases are not consistent (12–14). In addition, whether the excess risk of cardiovascular diseases linked with GDM is attributable to subsequent type 2 diabetes has not been fully clarified (10, 12). While several studies found that the association between GDM and cardiovascular disease is independent of intercurrent type 2 diabetes (7, 14), subsequent type 2 diabetes partly explained the increased risk of cardiovascular disease linked with GDM in a recent large population-based cohort study (12). Therefore, in this study, we aimed to explore the associations between a history of GDM and type-specific cardiovascular diseases, and assess the potential impact of type 2 diabetes on the associations, using data from the National Health and Nutrition Examination Survey (NHANES), 2007–2018. In addition, we also combined our results with those from previously related publications with a random-effect model.

## Materials and Methods

### Study Populations

The NHANES examines a nationally representative sample of about 5,000 persons each year, and the sample represents the non-institutionalized civilian population residing in counties across the United States. The sample design consists of multi-year, stratified, clustered four-stage samples, and data are released in 2-year cycles. Combination of 2 or more 2-year cycles is also a nationally representative sample (15). Data from NHANES have been widely used to determine the prevalence of major diseases and risk factors for diseases (15). The recent six NHANES 2-year cycles (2007/2008 to 2017/2018) specifically provided information for a history of GDM, and thus were included in this analysis.

Women fulfilling the criteria are included: (1) responding to the questions regarding a history of GDM; (2) with at least one live birth; (3) aged 20 years or older; and (4) responding to the questions regarding the presence of type-specific cardiovascular diseases. Women were excluded from the analysis if they were diagnosed with diabetes or type-specific cardiovascular diseases prior to a diagnosis of GDM. Finally, a total of 12,025 women were included in this analysis.

Previously observational studies with the exposure of interest as GDM and the outcomes of interest as type-specific cardiovascular diseases were included. However, studies only providing the results on GDM and overall cardiovascular diseases were excluded from this analysis, because the association between GDM and overall cardiovascular diseases has been addressed in a previous meta-analysis (7).

### History of GDM and Type-Specific Cardiovascular Diseases

A history of GDM was identified based on the question, “During your pregnancy, were you ever told by a doctor or other health professional that you had diabetes, sugar diabetes or gestational diabetes?”, and “How old were you when you were first told you had diabetes during a pregnancy?”. Women who answered yes to the question were considered to have a history of GDM (16, 17).

Coronary heart disease (CHD), heart failure and stroke were the outcomes of interest, and were self-reported through the following questions: “Has a doctor or other health professional ever told you that you had (1) coronary heart disease? (2) heart attack? (3) angina/angina pectoris? (4) congestive heart failure? (5) stroke?” and “How old were you when you were first told you had (1) coronary heart disease? 2) heart attack? (3) angina/angina pectoris? (4) congestive heart failure? (5) stroke?” In this analysis, women were identified to develop CHD if they answered reported having a diagnosis of coronary heart disease, heart attack, angina/angina pectoris, or congestive heart failure.

### Covariates

The following variables were included as covariates: age (continuous), race/ethnicity (Mexican–American, Other Hispanic, Non-Hispanic White, Non-Hispanic Black, Other Races), body mass index (<25 kg/m^2^, 25 to <30 kg/m^2^, ≥30 kg/m^2^), education (≤high school, some college or AA degree, ≥college graduate), annual family income (< $20,000, $20,000-$44,999, $45,000-$74,999, ≥$75,000), smoking (never smoker, former smoker, current smoker), alcohol drinking, recreational physical activity (vigorous/moderate recreational activities for at least 10 min continuously in a typical week), and daily intakes of energy, fat, fiber, vitamin C, vitamin B6 and vitamin D. The body measures data were collected by trained health technicians in the Mobile Examination Center. Demographics, smoking, alcohol and physical activity questionnaires were asked, in the home, by trained interviewers using the Computer-Assisted Personal Interview system.

### Diabetes, Hypertension, and Metabolic Syndrome

Women were identified to develop diabetes if they reported having a diagnosis of diabetes (other than during pregnancy) or, the hemoglobin A1c level was ≥6.5%, fasting plasma glucose level ≥126 mg/dL, or 2-h plasma glucose ≥200 mg/dL if diabetes was not diagnosed previously (18). Women were identified to develop type 1 diabetes if their age at diagnosis was <30 years and they are currently taking insulin (16). Metabolic syndrome was defined if there are any 3 of the 5 following metabolic-related disorders (19): elevated fasting glucose (≥100 mg/dL), elevated triglycerides (≥150 mg/dL), reduced HDL-C (<40 mg/dL in men, <50 mg/dL in women), elevated waist circumference (≥102 cm in men, ≥88 cm in women), and elevated blood pressure (≥130 mm Hg systolic blood pressure, ≥85 mm Hg diastolic blood pressure, mean values of three measurements). Women were identified to develop hypertension if they are currently taking antihypertensive medication, if systolic blood pressure was ≥130 mmHg, or if diastolic blood pressure was ≥80 mmHg (mean values of three measurements) (20).

### Statistical Analysis

Odds ratios (95% confidence interval) [OR (95% CI)] for type-specific cardiovascular diseases were calculated for women with a history of GDM compared to those without GDM. Sample weights, strata, and primary sampling units were used in logistic regression to account for the complex sample design of NHANES. Four different logistic regression models were calculated. Demographic variables including age and race/ethnicity were considered in model 1. Model 2 was further adjusted for body mass index and socioeconomic status (annual family income and education). Model 3 included the variables of model 2 with additional adjustment for health behaviors including alcohol drinking, smoking, and recreational physical activity. Model 4 included the variables of model 3 with additional adjustment for dietary factors (energy, fat, fiber, vitamin C, vitaminB6, and vitamin D). In order to determine the potential impact of individual chronic conditions (type 2 diabetes, hypertension and metabolic syndrome) on the risks of type-specific cardiovascular diseases conferred by GDM, we calculated the excess odds of type-specific cardiovascular diseases conferred by individual chronic conditions as [(OR_base – OR_adjusted)/(OR_base-1)] × 100 (12, 21). OR_base was derived from model 4, and OR_adjusted was further adjusted for individual chronic conditions of diabetes, metabolic syndrome, and hypertension, respectively. The 2-year sample weights were divided by 6 to compute the new multi-year sample weight, because 6 NHANES 2-year cycles were included in this analysis. Study-specific logarithms of risk estimates of type-specific cardiovascular diseases were combined with a random-effects model, which considers both between-study and within-study variation. *I*^2^ statistic was used to assess the between-study heterogeneity (22). Study quality was assessed using the 9-star Newcastle-Ottawa Scale, and a sensitivity analysis was conducted restricting to studies with higher quality scores (≥7 stars). Stata 12.0 was used in this study, and the analysis was considered significant if the corresponding *P*-value was ≤0.05.

## Results

### Population Characteristics

The weighted prevalence was 8.00% for GDM, 6.74% for CHD, 3.76% for stroke, and 2.73% for heart failure, respectively. Compared with women without GDM, women with a history of GDM tended to be younger (*P* < 0.01), more obese (*P* < 0.01), engage in more recreational physical activity (*P* = 0.05), and show higher prevalence of type 2 diabetes mellitus (*P* < 0.01) and metabolic syndrome (*P* < 0.01). Distributions of race/ethnicity (*P* < 0.01) and annual family income (*P* < 0.01) also differed significantly between the two groups, while there were no significant differences in smoking (*P* = 0.29) and alcohol drinking (*P* = 0.76). Table 1 presents the population characteristics of the participants according to the presence or absence of a history of GDM.

**Table 1:** Characteristics of the 2007–2018 NHANES adults according to the presence or absence of a history of gestational diabetes mellitus (GDM).

**Variables**	**Women with GDM (926)**	**Women without GDM (11,099)**	***P^a^***
Age [years, mean (SD)]	45.45 (12.31)	53.49 (16.76)	<0.01
Race/ethnicity (%)			<0.01
Mexican American	21.92	15.77	
Other Hispanic	11.34	11.69	
Non-Hispanic White	35.75	40.76	
Non-Hispanic Black	17.71	22.12	
Other race	13.28	9.67	
Annual family income (%)			<0.01
< $20 000	20.79	26.66	
$20 000–$34 999	33.45	33.70	
$35 000–$74 999	18.87	17.57	
≥$75,000	26.89	22.07	
Education (%)			<0.01
≤High school	43.74	50.15	
Some college or AA degree	36.07	30.64	
≥College graduate	20.19	19.20	
Vigorous/moderate recreational activities for at least 10 min continuously in a typical week (%)	44.04	40.84	0.05
Smoking			0.29
Current smoker	18.90	17.46	
Former smoker	17.82	19.65	
Never smoker	63.28	62.89	
Body mass index (%)			<0.01
<25 kg/m^2^	18.33	27.26	
25–29 kg/m^2^	25.70	29.66	
≥30 kg/m^2^	55.97	43.08	
Alcohol [g/day, mean (SD)]	4.18 (16.56)	4.04 (12.77)	0.76
Type 2 diabetes (%)	34.99	17.83	<0.01
Hypertension (%)	47.95	56.25	<0.01
Metabolic syndrome (%)	49.68	40.19	<0.01

*M, Mean values, SD, standard deviation*.

a *t-test was performed for continuous variables, and Chi-square test was performed for categorical variables*.

We identified 11 studies (5 prospective cohort studies, 5 retrospective cohort studies, 1 cross-sectional study) on GDM and the risks of type-specific cardiovascular diseases (Table 2). Therefore, there are a total of 12 studies (including our study) in the combined analysis on GDM and CHD (12 studies, 7,615,322 participants), heart failure (5 studies, 4,491,665 participants), and cerebrovascular disease (9 studies, 6,090,848 participants) in this analysis.

**Table 2:** Included studies on the associations between gestational diabetes mellitus and type-specific cardiovascular diseases.

**Reference**	**Study design, age**	**Follow-up years**	**No. of participants**	**No. of cases**	**Outcomes**	**Risk estimates (95% CIs)**	**Impact of T2DM on the findings**
Yu et al. (12), Denmark	Prospective cohort study, Parous women (≥18 years at baseline)	16.2	1,002,486	24,045 17,347 3,888	CHD Cerebrovascular disease Heart failure	2.02 (1.85–2.21) 1.47 (1.30–1.67) 2.20 (1.76–2.74)	T2DM accounts for 25.0–38.3% (CHD), 2.1% (cerebrovascular disease) and 64.2% (heart failure) of the elevated risks associated with GDM.
Sun et al. (13), Korea]	Retrospective cohort study, 20–49 years	12.8	1,500,168	12,698 8,890 2,367	CHD Cerebrovascular disease Heart failure	1.26 (1.05–1.51) 1.04 (0.98–1.11) 1.20 (1.07–1.35)	The associations with type- specific cardiovascular diseases were much stronger in women with both GDM and T2DM.
Gunderson et al. (14), USA	Prospective cohort study, 18–30 at baseline	25	1,133	183	CHD	1.66 (1.13–2.42)	Levels of subsequent glucose tolerance did not influence the results materially. However, the association was only significant in women without diabetes.
Echouffo-Tcheugui et al. (23), Canada	Prospective cohort study, Mean age: 30 years at baseline	7	906,319	763	Heart failure	1.62 (1.28–2.05)	The association was attenuated after further adjustment for other chronic diseases including diabetes.
Perera et al. (24), USA	Cross-sectional study, 20–73 years	–	8,262	93	CHD	1.6 (0.8–2.8)	–
McKenzie-Sampson et al. (25), Canada	Retrospective cohort study, mean age: ~28 years at baseline	A maximum of 25.2 years	1,070,667	4,736 1,430 3,781	CHD Heart failure Stroke	2.16 (1.95–2.39) 2.00 (1.66–2.42) 1.41 (1.23–1.61)	–
Daly et al. (26), UK	Retrospective cohort study, <50 years	–	46,399	9,112 9,106	CHD Cerebrovascular disease	2.78 (1.37–5.66) 0.95 (0.51–1.77)	–
Tobias et al. (27), USA	Prospective cohort study, 24–44 years at baseline	25.7	89,479	612 553	CHD Stroke	1.45 (1.05–1.99) 1.10 (0.75–1.61)	Compared with women without diabetes, women with T2DM only, or both GDM and T2DM had a 4-fold elevated risk of CHD and 3-fold elevated risk of stroke. The association was not significant in women with a history of GDM but without progression to T2DM.
Retnakaran et al. (28), Canada	Prospective cohort study, Median age: 31 years	10.0	1,515,079	–	CHD	2.56 (2.21–2.95)	Among women who had GDM, the hazard ratio of CHD was much higher for women who also developed T2DM [3.54 (2.96–4.23)] than women who did not develop T2DM [1.41 (1.11–1.80)].
Goueslard et al. (29), France	Retrospective cohort study, Median age: 29 years	7	1,518,990	930 1,252	CHD Stroke	1.77 (1.43–2.18) 1.28 (1.01–1.62)	Among women who had GDM, the odds ratio of CHD was much higher for women who also developed T2DM [5.45 (2.38–12.45)] than women who did not develop T2DM [1.92 (1.36–2.71)].
Savitz et al. (30), USA	Retrospective cohort study, –	1	849,639	81 126	CHD Cerebrovascular disease	1.5 (0.7–3.1) 1.2 (0.7–2.3)	–
Carr et al. (31), USA	Cross-sectional study, 51.1	–	995	–	CHD Stroke	1.58 (1.00–2.49) 1.67 (0.87–3.22)	–

*CHD, coronary heart disease, T2DM, type 2 diabetes*.

### Association of GDM With CHD

Overall, the findings on a history of GDM and CHD were similar across the 4 different logistic regression models. In model 4, compared with women without GDM, the multivariable-adjusted OR (95% CI) for CHD were 1.82 (1.21–2.72) among women with a history of GDM. Further adjustment for hypertension and metabolic syndrome did not change the results materially. However, the association was attenuated after further adjustment for type 2 diabetes [1.46 (0.99–2.15)] (Table 3). The analysis showed that type 2 diabetes, hypertension and metabolic syndrome explained 43.90, 1.22, and 3.66% of the excess odds of CHD associated with GDM, respectively.

**Table 3:** Odds ratios of coronary heart disease, heart failure and stroke for women with a history of gestational diabetes mellitus compared with those without a history of gestational diabetes mellitus.

**Groups**		**Odds ratios (95% confidence intervals)**						
	**Cases/N**	**Model 1**	**Model 2**	**Model 3**	**Model 4**	**Model 4 +**	**Model 4 +**	**Model 4 +**
						**hypertension**	**MetS**	**Type 2 diabetes**
CHD	933/12,025	1.93 (1.25–2.97)^**^	1.82 (1.25–2.65)^**^	1.80 (1.21–2.67)^**^	1.82 (1.21–2.72)^**^	1.81 (1.21–2.69)^**^	1.79 (1.21–2.66)^**^	1.46 (0.99–2.15)
Heart failure	396/11,604	1.40 (0.79–2.47)	1.40 (0.80–2.46)	1.41 (0.80–2.49)	1.43 (0.80–2.53)	1.41 (0.79–2.50)	1.42 (0.80–2.53)	1.14 (0.64–2.05)
Stroke	536/12,025	1.41 (0.84–2.37)	1.20 (0.79–1.82)	1.19 (0.76–1.84)	1.19 (0.76–1.86)	1.19 (0.76–1.86)	1.19 (0.76–1.85)	1.07 (0.67–1.69)

** *P < 0.01*.

*Model 1: adjusted for age and race/ethnicity*.

*Model 2: adjusted for covariates in model 1 and body mass index, education, and annual family income*.

*Model 3: adjusted for covariates in model 2 and alcohol drinking, smoking, and recreational physical activity*.

*Model 4: adjusted for covariates in model 3 and dietary factors (energy, fat, fiber, vitamin C, vitamin B6 and vitamin D)*.

### Associations of GDM With Heart Failure and Stroke

Women with a history of GDM had increased but not significant odds of heart failure [1.43 (0.80–2.53)] and stoke [1.19 (0.76–1.86)] compare with women without GDM, which might be caused by the relatively wide ranges of 95% CIs (Table 3). The analysis showed that type 2 diabetes, hypertension and metabolic syndrome explained 67.44, 4.65, and 2.33% of the excess odds of heart failure associated with GDM, respectively, and the figures were 63.16, 0.00, 0.00% for stroke.

### Previously Related Studies

**Figure 1:**
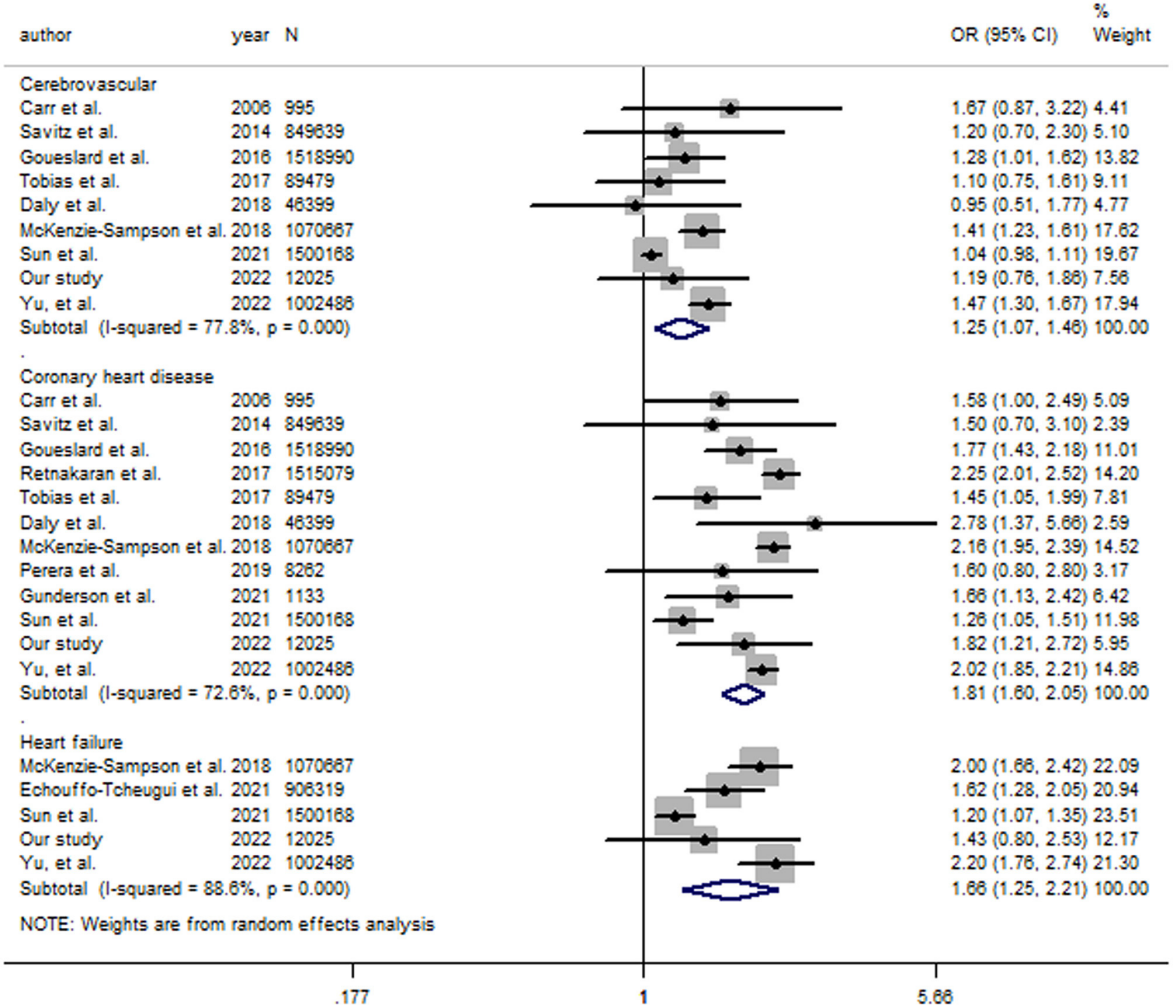
The forest plot for gestational diabetes mellitus and risks of coronary heart disease, heart failure and cerebrovascular disease. The size of gray box is positively proportional to the weight assigned to each study, which is inversely proportional to the standard error of the OR, and horizontal lines represent the 95 % confidence intervals. OR (95% CI): Odds ratio (95% confidence interval).

Among the 11 previously related studies, GDM was positively associated with the risk of CHD in 9 studies, while the association was statistically not significant in the other two studies (Table 2). GDM was associated with an increased risk of heart failure in all of the 4 studies (Table 2). A significant association was found between GDM and cerebrovascular disease in 3 studies, while the association was statistically not significant in the other 5 studies (Table 2). The random-effect model combining results from this study with those from previously related studies yielded ORs (95% CIs) of 1.81 (1.60–2.05) for CHD (*I*^2^= 72.6%), 1.66 (1.25–2.21) for heart failure (*I*^2^= 88.6%), and 1.25 (1.07–1.46) for cerebrovascular disease (*I*^2^= 77.8%) (Figure 1). In a sensitivity analysis, the combined results from studies with higher quality scores (≥7 stars) were 1.83 (1.59–2.10) for CHD [9 studies (12–14, 25–30), 7,594,040 participants], 1.70 (1.24–2.32) for heart failure [4 studies (12, 13, 23, 25), 4,479,640 participants], and 1.24 (1.04–1.47) for cerebrovascular disease [7 studies (12, 13, 25–27, 29, 30), 6,077,828 participants].

## Discussion

Results from this nationally representative survey cohort showed that GDM had stronger associations with coronary heart diseases and heart failure than cerebrovascular disease, and the excess risks are attributable, in part, to type 2 diabetes. In the analysis combing results from previously related studies, we observed 81% higher odds of developing CHD, 66% higher odds of developing heart failure, and 25% higher odds of developing cerebrovascular disease for women with a history of GDM compared with women without GDM.

The biological plausibility for causality in that development of GDM can promote cardiovascular diseases are as follows: First, GDM could increase the risk of long-term complications including type 2 diabetes, metabolic syndrome and hypertension (2–6), which are detrimental to cardiovascular health. In particular, women with a history of GDM have a nearly 10-fold higher risk of developing type 2 diabetes than women without a history of GDM (2). Diabetes showed stronger associations with ischemic heart disease [relative risk (95% CI): 2.46 (2.39–2.53)] and other forms of heart disease [1.98 (1.88–2.08)] than cerebrovascular disease [1.70 (1.61–1.80) in women in US adults (32). Second, in patients with GDM, the underlying metabolic defects including chronic beta-cell dysfunction and insulin resistance could increase levels of glycemia, LDL cholesterol, blood pressure and adiposity, but decrease HDL cholesterol levels, which in together increase the risks of CHD, stroke and heart failure (8). Abnormal expression of cardiovascular diseases associated microRNAs was observed 3–11 years after delivery in women with a history of GDM (33). In addition, less favorable profiles of circulating inflammatory markers including tumor necrosis factor-α, C-reactive protein, and adiponectin in patients with GDM may also contribute to the association between a history of GDM and risk of cardiovascular diseases (34). Third, hyperglycemia exposure even in a short period could significantly induce functional cardiac impairment in women with GDM (35). In addition, a history of GDM was associated with a 2-fold higher risk of coronary artery calcification, and the association was independent of other traditional risk factors of cardiovascular diseases (14), suggesting there is a direct association between GDM itself and the development of cardiovascular diseases (10). Fourth, according to the Barker's Hypothesis, epigenetic factors may predispose to the development of cardiovascular events in offspring (36, 37). A previous meta-analysis found that offspring born to mothers with GDM have elevated systolic blood pressure, glucose and body mass index (38), and population based cohort studies also showed an elevated risk of cardiovascular disease in offspring exposed to GDM (39–41).

However, data from the 2007–2014 NHANES showed that a history of GDM was only associated with lower HDL cholesterol, and the associations with systolic or diastolic blood pressure, total cholesterol, triglycerides, or LDL-cholesterol were not significant (17). These findings indicate that HDL cholesterol maybe a key factor in the association between a history of GDM and future development of cardiovascular diseases. A recent meta-analysis of 32 prospective cohort studies found that the death risk from cardiovascular diseases was reduced by 23% (95% CI: 13–31%) with each 1 mmol/L increment in HDL cholesterol levels (42). In addition, both high levels of cholesterol efflux capacity, antioxidant capacity, and anti-inflammatory capacity of HDL were associated with lower cardiovascular disease risk, while further studies are still needed to confirm these findings (43). However, HDL subspecies defined by the components of minor protein or lipid could exert diverse effects on the development of cardiovascular disease (44). While atheroprotective effects of HDL containing APOE or APOC1 were observed (45, 46), APOC-III-containing HDL was associated with higher carotid intima-media thickness (47) and higher risk of CHD (48) in the general population. In addition, HDL subspecies containing haptoglobin, complement C3, alpha-2 macroglobulin, or plasminogen were also associated with higher risk of CHD (45). These findings highlights the need for further studies on the associations between a history of GDM and levels of HDL subspecies in later life. In our study, the associations between a history of GDM and type-specific cardiovascular diseases remained unchanged after further adjustment for hypertension and metabolic syndrome. Our results are consistent with those from the previous study (17) showing that hypertension and metabolic syndrome do not contribute to the association between a history of GDM and type-specific cardiovascular diseases.

In this study, we found that type 2 diabetes was associated with 43.90, 67.44, and 63.16% of the excess odds of CHD, heart failure and stroke associated with GDM, respectively. A previous meta-analysis found that a history of GDM was associated with 2-fold higher risk of total cardiovascular disease [1.98 (1.57–2.50)], and incidence of type 2 diabetes did not impact the association between GDM and total cardiovascular disease in univariate meta-regression (7). However, an attenuated but significant association was found among women without type 2 diabetes [1.56 (1.04–2.32)] (7). Several recent studies have also assessed the role of intercurrent type 2 diabetes on the risk of cardiovascular disease associated with GDM. A recent large population-based prospective cohort study including 10,02,486 Danish women showed that type 2 diabetes was associated with 25.0, 64.2, and 10.1% of the excess odds of CHD, heart failure and stroke associated with GDM, respectively, suggesting that the excess risks could be partly explained by subsequent type 2 diabetes (12). Another recent large population-based retrospective cohort study including 1,500,168 Korean women found that, compared to women without GDM or type 2 diabetes, an increased risk of total CVD was observed for women who had GDM and developed type 2 diabetes during follow-up [1.74 (1.40–21.5)], while the association was not statistically significant for women with GDM only [1.06 (1.00–1.12)], suggesting that type 2 diabetes accounts for much of the excess risk (13). In addition, a population-based prospective cohort study of 1,515,079 women conducted in Canada also found stronger associations between GDM and total cardiovascular disease and CHD in women who had a history of GDM and developed type 2 diabetes during follow-up [2.82 (2.41–3.30) for total cardiovascular disease and 3.54 (0.96–4.23) for CHD] than women who had a history of GDM but did not develop type 2 diabetes during follow-up [1.30 (1.07–1.59) for total cardiovascular disease and 1.41 (1.11–1.80) for CHD] (28). In summary, the recent findings from different countries suggested that type 2 diabetes partly explains the increased risk of cardiovascular disease linked with GDM.

High between-study heterogeneity (*I*^2^ statistic) was found in the analysis between GDM and CHD, heart failure and cerebrovascular disease, respectively. However, direction of the associations between GDM and CHD, heart failure and cerebrovascular disease are generally consistent among the included studies, and the high between-study heterogeneity could be caused by the larger risk estimates and narrow 95% CIs in several large population-based cohort studies. As shown in Figure 1, the magnitude of the association between a history of GDM and CHD was apparently larger in several of the large population-based cohort studies with long follow-up period [the study by McKenzie-Sampson et al. (25), *N* = 1,070,667, a follow-up of up to 25.2 years: 2.16 (1.95–2.39); the study by Retnakaran et al. (28), *N* = 1,515,079, a median follow-up of 10.0 years: 2.56 (2.21–2.95); the study by Yu et al. (12), *N* = 1,002,486, a median follow-up of 16.2 years: 2.02 (1.85–2.21)]. The association between a history of GDM and heart failure was also more pronounced in several of the large population-based cohort studies with long follow-up period [the study by McKenzie-Sampson et al. (25), *N* = 1,070,667, a follow-up of up to 25.2 years: 2.00 (1.66–2.42); the study by Yu et al. (12), *N* = 1,002,486, a median follow-up of 16.2 years: 2.20 (1.76–2.74)]. A stronger association was also found between a history of GDM and cerebrovascular disease in several of the large population-based cohort studies with long follow-up period [the study by McKenzie-Sampson et al. (25), *N* = 1,070,667, a follow-up of up to 25.2 years: 1.41 (1.23–1.61); the study by Yu et al. (12). *N* = 1,002,486, a median follow-up of 16.2 years: 1.47 (1.30–1.67)]. In summary, the findings available support the associations between a history of GDM and future risk of type-specific cardiovascular diseases, and the associations are more pronounced in large population-based cohort studies with long follow-up period.

There are several strengths in this study. This is a nationally representative survey cohort, and we also combined our results with those from previously related studies. In addition, we also found type 2 diabetes could partly explain the increased odds of type-specific cardiovascular disease linked with GDM. However, there are also several limitations. First, causality between GDM and risks of type-specific cardiovascular diseases cannot be determined in this study. However, we have excluded women who were diagnosed with type-specific cardiovascular diseases prior to a diagnosis of GDM from the analysis, and the positive associations between a history of GDM and type-specific cardiovascular diseases were also observed in previously prospective cohort studies. Second, there may be misclassification because a history of GDM and diagnosis of type-specific cardiovascular diseases were based on self-report. However, data from NHANES have been widely used to determine the prevalence of major diseases and risk factors for diseases (15), and the risk estimates in this analysis are generally comparable to the combined results from previously related studies. In addition, non-differential misclassification could have weakened an association. Other information including control of the glycaemia levels during the three trimesters of pregnancy are not available in our study and are also missing in the included previous studies on GDM history and risk of type-specific cardiovascular diseases, which should be considered in further studies. Third, although we have adjusted for a number of potential covariates, residual confounding arising from other unmeasured variables could be of concern. However, as shown in Table 3, the associations between a history of GDM and type-specific cardiovascular diseases did not change materially across the four statistical models, suggesting the associations were independent of these covariates and supporting a direct association between GDM itself and future development of type-specific cardiovascular diseases.

Patients with GDM require anti-diabetic pharmacotherapy if the glycaemia levels cannot be maintained with diet modification, and insulin and metformin are recommended for the treatment of GDM (49–51). Results from two recent meta-analysis of randomized controlled trials suggested that metformin treatment could reduce the further risk of cardiovascular diseases (52, 53). However, over a 21-year median follow-up, neither metformin nor lifestyle interventions could reduce the risks of myocardial infarction and stroke in the DPP/DPPOS (54) (the longest and largest trial of metformin treatment for diabetes prevention). In addition, baseline metformin treatment did not provide additional cardioprotective effect associated with dulaglutide in another large trail (55). For insulin, two recent meta-analysis of randomized clinical trials showed that baseline insulin treatment was not associated with further risks of cardiovascular events or death (56, 57). As the first-line treatment, nutritional interventions have made recommendations on intakes of carbohydrate, fat, and protein (58). Although most of these clinical practice guidelines on nutritional interventions are not being of high quality (58), the nutritional interventions could significantly reduce the risk of postpartum diabetes (59). However, evidence is limited regarding diet modification and anti-diabetic pharmacotherapy including metformin treatment during pregnancy and risks of cardiovascular diseases in women with a history of GDM (60). Information for insulin, metformin and diet modification during pregnancy are not available in our study. The long-term effects of these anti-diabetic pharmacotherapies and diet modification during pregnancy on the further risks of cardiovascular diseases among women with a history of GDM deserve to be determined further.

Results from our analysis and previous studies showed that GDM provides unique opportunities for identifying women at increased risks of type-specific cardiovascular diseases (9). For the primary prevention of cardiovascular disease including CHD, stroke and heart failure, the recent Statement From the American Heart Association (61) recommends recognizing GDM when evaluating risk of cardiovascular disease, increasing physical activity, adopting a heart-healthy diet, lactation and breastfeeding, and calls for future studies of pharmacotherapy including metformin among women who previously had GDM.

In summary, data from the NHANES showed that GDM had stronger associations with CHD and heart failure than cerebrovascular disease, and the excess risks are attributable, in part, to type 2 diabetes. Combined results from this analysis with those from previously related studies showed that a history of GDM was associated with 81% higher risk of CHD, 66% higher risk of heart failure, and 25% higher risk of cerebrovascular disease.

## Data Availability Statement

Publicly available datasets were analyzed in this study. This data can be found here: The datasets generated during and/or analyzed during the current study are available in the NHANES: https://www.cdc.gov/nchs/nhanes/.

## Ethics Statement

NHANES was approved by the National Center for Health Statistics Research Ethics Review Board. The patients/participants provided their written informed consent to participate in this study.

## Author Contributions

YM and QL designed the study. WH conducted the statistical analysis. YM, BX, LL, XH, and QL drafted the manuscript. QL made critical revisions. All authors have approved the final article.

## Funding

The authors receive support from the Maternal and Child Health Research Project of Jiangsu Province (No. F201720) and the Development Science and Technology Project of Kunshan (No. KS1646).

## Conflict of Interest

The authors declare that the research was conducted in the absence of any commercial or financial relationships that could be construed as a potential conflict of interest.

## Publisher's Note

All claims expressed in this article are solely those of the authors and do not necessarily represent those of their affiliated organizations, or those of the publisher, the editors and the reviewers. Any product that may be evaluated in this article, or claim that may be made by its manufacturer, is not guaranteed or endorsed by the publisher.

## References

[1] Wang H Li N Chivese T Werfalli M Sun H Yuen L. IDF diabetes atlas: estimation of global and regional gestational diabetes mellitus prevalence for 2021 by International Association of Diabetes in Pregnancy Study Group's Criteria. Diabetes Res Clin Pract. (2022) 183:109050. 10.1016/j.diabres.2021.109050 34883186

[2] Vounzoulaki E Khunti K Abner SC Tan BK Davies MJ Gillies CL. Progression to type 2 diabetes in women with a known history of gestational diabetes: systematic review and meta-analysis. BMJ. (2020) 369:m1361. 10.1136/bmj.m1361 32404325 PMC7218708

[3] Dennison RA Chen ES Green ME Legard C Kotecha D Farmer G. The absolute and relative risk of type 2 diabetes after gestational diabetes: a systematic review and meta-analysis of 129 studies. Diabetes Res Clin Pract. (2021) 171:108625. 10.1016/j.diabres.2020.108625 33333204 PMC7610694

[4] Tranidou A Dagklis T Tsakiridis I Siargkas A Apostolopoulou A Mamopoulos A. Risk of developing metabolic syndrome after gestational diabetes mellitus - a systematic review and meta-analysis. J Endocrinol Invest. (2021) 44:1139–49. 10.1007/s40618-020-01464-6 33226626

[5] Pathirana MM Lassi Z Ali A Arstall M Roberts CT Andraweera PH. Cardiovascular risk factors in women with previous gestational diabetes mellitus: a systematic review and meta-analysis. Rev Endocr Metab Disord. (2021) 22:729–61. 10.1007/s11154-020-09587-0 33106997

[6] McIntyre HD Catalano P Zhang C Desoye G Mathiesen ER Damm P. Gestational diabetes mellitus. Nat Rev Dis Primers. (2019) 5:47. 10.1038/s41572-019-0098-8 31296866

[7] Kramer CK Campbell S Retnakaran R. Gestational diabetes and the risk of cardiovascular disease in women: a systematic review and meta-analysis. Diabetologia. (2019) 62:905–14. 10.1007/s00125-019-4840-2 30843102

[8] Fu J Retnakaran R. The life course perspective of gestational diabetes: an opportunity for the prevention of diabetes and heart disease in women. EClinicalMedicine. (2022) 45:101294. 10.1016/j.eclinm.2022.101294 35198924 PMC8850315

[9] Saravanan P. Gestational diabetes: opportunities for improving maternal and child health. Lancet Diabetes Endocrinol. (2020) 8:793–800. 10.1016/S2213-8587(20)30161-3 32822601

[10] Green JB. Cardiovascular consequences of gestational diabetes. Circulation. (2021) 143:988–90. 10.1161/CIRCULATIONAHA.120.052995 33683943

[11] Management of diabetes in pregnancy: standards of medical care in diabetes-2021. Diabetes Care. (2021) 44:S200–10. 10.2337/dc21-S014 33298425

[12] Yu Y Soohoo M Sorensen HT Li J Arah OA. Gestational diabetes mellitus and the risks of overall and type-specific cardiovascular diseases: a population- and sibling-matched cohort study. Diabetes Care. (2022) 45:151–9. 10.2337/dc21-1018 34764208 PMC8753767

[13] Sun J Kim GR Lee SJ Kim HC. Gestational diabetes mellitus and the role of intercurrent type 2 diabetes on long-term risk of cardiovascular events. Sci Rep. (2021) 11:21140. 10.1038/s41598-021-99993-4 34707209 PMC8551203

[14] Gunderson EP Sun B Catov JM Carnethon M Lewis CE Allen NB. Gestational diabetes history and glucose tolerance after pregnancy associated with coronary artery calcium in women during midlife: the CARDIA study. Circulation. (2021) 143:974–87. 10.1161/CIRCULATIONAHA.120.047320 33517667 PMC7940578

[15] Centers for Disease Control and Prevention. Available online at: https://www.cdc.gov/nchs/nhanes/about_nhanes.htm (accessed March 23, 2022).

[16] Ciardullo S Bianconi E Zerbini F Perseghin G. Current type 2 diabetes, rather than previous gestational diabetes, is associated with liver disease in U.S. Women. Diabetes Res Clin Pract. (2021) 177:108879. 10.1016/j.diabres.2021.108879 34058299

[17] Shostrom DCV Sun Y Oleson JJ Snetselaar LG Bao W. History of gestational diabetes mellitus in relation to cardiovascular disease and cardiovascular risk factors in US women. Front Endocrinol. (2017) 8:144. 10.3389/fendo.2017.00144 28694789 PMC5483836

[18] Menke A Casagrande S Geiss L Cowie CC. Prevalence of and trends in diabetes among adults in the United States, 1988-2012. JAMA. (2015) 314:1021–9. 10.1001/jama.2015.10029 26348752

[19] Grundy SM Cleeman JI Daniels SR Donato KA Eckel RH Franklin BA. Diagnosis and management of the metabolic syndrome: an American Heart Association/National Heart, Lung, and Blood Institute Scientific Statement. Circulation. (2005) 112:2735–52. 10.1161/CIRCULATIONAHA.105.169404 16157765

[20] Whelton PK Carey RM Aronow WS Casey DE Jr Collins KJ Dennison Himmelfarb C. 2017 ACC/AHA/AAPA/ABC/ACPM/AGS/APhA/ASH/ASPC/NMA/PCNA Guideline for the Prevention, Detection, Evaluation, and Management of High Blood Pressure in Adults: Executive Summary: a report of the American College of Cardiology/American Heart Association Task Force on Clinical Practice Guidelines. J Am Coll Cardiol. (2018) 71:2199–269. 10.1161/HYP.0000000000000075 29146533

[21] Kalyani RR Saudek CD Brancati FL Selvin E. Association of diabetes, comorbidities, and A1C with functional disability in older adults: results from the National Health and Nutrition Examination Survey (NHANES), 1999-2006. Diabetes Care. (2010) 33:1055–60. 10.2337/dc09-1597 20185736 PMC2858174

[22] Higgins JP Thompson SG Deeks JJ Altman DG. Measuring inconsistency in meta-analyses. BMJ. (2003) 327:557–60. 10.1136/bmj.327.7414.557 12958120 PMC192859

[23] Echouffo-Tcheugui JB Guan J Retnakaran R Shah BR. Gestational diabetes and incident heart failure: a cohort study. Diabetes Care. (2021) 44:2346–52. 10.2337/figshare.14999628.v1 34385145 PMC8929190

[24] Perera MJ Reina SA Elfassy T Potter JE Sotres Alvarez D Simon MA. Gestational diabetes and cardiovascular risk factors and disease in US Hispanics/Latinas in the Hispanic Community Health Study/Study of Latinos (HCHS/SOL). Women Health. (2019) 59:481–95. 10.1080/03630242.2018.1500415 30040600 PMC6536260

[25] McKenzie-Sampson S Paradis G Healy-Profitos J St-Pierre F Auger N. Gestational diabetes and risk of cardiovascular disease up to 25 years after pregnancy: a retrospective cohort study. Acta Diabetol. (2018) 55:315–22. 10.1007/s00592-017-1099-2 29327149

[26] Daly B Toulis KA Thomas N Gokhale K Martin J Webber J. Increased risk of ischemic heart disease, hypertension, and type 2 diabetes in women with previous gestational diabetes mellitus, a target group in general practice for preventive interventions: a population-based cohort study. PLoS Med. (2018) 15:e1002488. 10.1371/journal.pmed.1002488 29337985 PMC5770032

[27] Tobias DK Stuart JJ Li S Chavarro J Rimm EB Rich-Edwards J. Association of history of gestational diabetes with long-term cardiovascular disease risk in a large prospective cohort of US women. JAMA Intern Med. (2017) 177:1735–42. 10.1001/jamainternmed.2017.2790 29049820 PMC5820722

[28] Retnakaran R Shah BR. Role of Type 2 Diabetes in determining retinal, renal, and cardiovascular outcomes in women with previous gestational diabetes mellitus. Diabetes Care. (2017) 40:101–8. 10.2337/dc16-1400 27821407

[29] Goueslard K Cottenet J Mariet AS Giroud M Cottin Y Petit JM. Early cardiovascular events in women with a history of gestational diabetes mellitus. Cardiovasc Diabetol. (2016) 15:15. 10.1186/s12933-016-0338-0 26817691 PMC4728938

[30] Savitz DA Danilack VA Elston B Lipkind HS. Pregnancy-induced hypertension and diabetes and the risk of cardiovascular disease, stroke, and diabetes hospitalization in the year following delivery. Am J Epidemiol. (2014) 180:41–4. 10.1093/aje/kwu118 24879314 PMC4070939

[31] Carr DB Utzschneider KM Hull RL Tong J Wallace TM Kodama K. Gestational diabetes mellitus increases the risk of cardiovascular disease in women with a family history of type 2 diabetes. Diabetes Care. (2006) 29:2078–83. 10.2337/dc05-2482 16936156

[32] Campbell PT Newton CC Patel AV Jacobs EJ Gapstur SM. Diabetes and cause-specific mortality in a prospective cohort of one million U.S. adults. Diabetes Care. (2012) 35:1835–44. 10.2337/dc12-0002 22699290 PMC3425000

[33] Hromadnikova I Kotlabova K Dvorakova L Krofta L. Diabetes mellitus and cardiovascular risk assessment in mothers with a history of gestational diabetes mellitus based on postpartal expression profile of microRNAs associated with diabetes mellitus and cardiovascular and cerebrovascular diseases. Int J Mol Sci. (2020) 21:2437. 10.3390/ijms21072437 32244558 PMC7177375

[34] Sullivan SD Umans JG Ratner R. Gestational diabetes: implications for cardiovascular health. Curr Diab Rep. (2012) 12:43–52. 10.1007/s11892-011-0238-3 22037824

[35] Buddeberg BS Sharma R O'Driscoll JM Kaelin Agten A Khalil A Thilaganathan B. Impact of gestational diabetes mellitus on maternal cardiac adaptation to pregnancy. Ultrasound Obstet Gynecol. (2020) 56:240–6. 10.1002/uog.21941 31785176

[36] Barker DJ. The origins of the developmental origins theory. J Intern Med. (2007) 261:412–7. 10.1111/j.1365-2796.2007.01809.x 17444880

[37] Barker DJ. Fetal origins of coronary heart disease. BMJ. (1995) 311:171–4. 10.1136/bmj.311.6998.171 7613432 PMC2550226

[38] Pathirana MM Lassi ZS Roberts CT Andraweera PH. Cardiovascular risk factors in offspring exposed to gestational diabetes mellitus *in utero*: systematic review and meta-analysis. J Dev Orig Health Dis. (2020) 11:599–616. 10.1017/S2040174419000850 31902382

[39] Guillemette L Wicklow B Sellers EAC Dart A Shen GX Dolinsky VW. Intrauterine exposure to diabetes and risk of cardiovascular disease in adolescence and early adulthood: a population-based birth cohort study. CMAJ. (2020) 192:E1104–13. 10.1503/cmaj.190797 32989023 PMC7532013

[40] Yu Y Arah OA Liew Z Cnattingius S Olsen J Sorensen HT. Maternal diabetes during pregnancy and early onset of cardiovascular disease in offspring: population based cohort study with 40 years of follow-up. BMJ. (2019) 367:l6398. 10.1136/bmj.l6398 31801789 PMC6891797

[41] Leybovitz-Haleluya N Wainstock T Landau D Sheiner E. Maternal gestational diabetes mellitus and the risk of subsequent pediatric cardiovascular diseases of the offspring: a population-based cohort study with up to 18 years of follow up. Acta Diabetol. (2018) 55:1037–42. 10.1007/s00592-018-1176-1 29936651

[42] Liu L Han M Qie R Li Q Zhang X Zhang J. A dose-response meta-analysis to evaluate the relationship between high-density lipoprotein cholesterol and all-cause and cardiovascular disease mortality. J Endocrinol Invest. (2022) 45:551–62. 10.1007/s40618-021-01690-6 34676492

[43] Soria-Florido MT Schroder H Grau M Fito M Lassale C. High density lipoprotein functionality and cardiovascular events and mortality: a systematic review and meta-analysis. Atherosclerosis. (2020) 302:36–42. 10.1016/j.atherosclerosis.2020.04.015 32438197

[44] Davidson WS Cooke AL Swertfeger DK Shah AS. The difference between high density lipoprotein subfractions and subspecies: an evolving model in cardiovascular disease and diabetes. Curr Atheroscler Rep. (2021) 23:23. 10.1007/s11883-021-00925-4 33772657 PMC8258923

[45] Sacks FM Liang L Furtado JD Cai T Davidson WS He Z. Protein-defined subspecies of hdls (high-density lipoproteins) and differential risk of coronary heart disease in 4 prospective studies. Arterioscler Thromb Vasc Biol. (2020) 40:2714–27. 10.1161/ATVBAHA.120.314609 32907368 PMC7577984

[46] Morton AM Furtado JD Mendivil CO Sacks FM. Dietary unsaturated fat increases HDL metabolic pathways involving apoE favorable to reverse cholesterol transport. JCI Insight. (2019) 4:e124620. 10.1172/jci.insight.124620 30944249 PMC6483656

[47] Yamamoto R Sacks FM Hu FB Rosner B Furtado JD Aroner SA. High density lipoprotein with apolipoprotein C-III is associated with carotid intima-media thickness among generally healthy individuals. Atherosclerosis. (2018) 269:92–9. 10.1016/j.atherosclerosis.2017.12.029 29351856

[48] Jensen MK Aroner SA Mukamal KJ Furtado JD Post WS Tsai MY. High-density lipoprotein subspecies defined by presence of apolipoprotein c-iii and incident coronary heart disease in four cohorts. Circulation. (2018) 137:1364–73. 10.1161/CIRCULATIONAHA.117.031276 29162611 PMC5871573

[49] Brown J Grzeskowiak L Williamson K Downie MR Crowther CA. Insulin for the treatment of women with gestational diabetes. Cochrane Database Syst Rev. (2017) 11:CD012037. 10.1002/14651858.CD012037.pub2 29103210 PMC6486160

[50] Balsells M Garcia-Patterson A Sola I Roque M Gich I Corcoy R. Glibenclamide, metformin, and insulin for the treatment of gestational diabetes: a systematic review and meta-analysis. BMJ. (2015) 350:h102. 10.1136/bmj.h102 25609400 PMC4301599

[51] Musa OAH Syed A Mohamed AM Chivese T Clark J Furuya-Kanamori L. Metformin is comparable to insulin for pharmacotherapy in gestational diabetes mellitus: a network meta-analysis evaluating 6046 women. Pharmacol Res. (2021) 167:105546. 10.1016/j.phrs.2021.105546 33716167

[52] Monami M Candido R Pintaudi B Targher G Mannucci E. Effect of metformin on all-cause mortality and major adverse cardiovascular events: An updated meta-analysis of randomized controlled trials. Nutr Metab Cardiovasc Dis. (2021) 31:699–704. 10.1016/j.numecd.2020.11.031 33549430

[53] Zhang K Yang W Dai H Deng Z. Cardiovascular risk following metformin treatment in patients with type 2 diabetes mellitus: results from meta-analysis. Diabetes Res Clin Pract. (2020) 160:108001. 10.1016/j.diabres.2020.108001 31904444

[54] Goldberg RB Orchard TJ Crandall JP Boyko EJ Budoff M Dabelea D. Effects of long-term metformin and lifestyle interventions on cardiovascular events in the diabetes prevention program and its outcome study. Circulation. (2022) 145:1632–41. 10.1161/CIRCULATIONAHA.121.056756 35603600 PMC9179081

[55] Ferrannini G Gerstein H Colhoun HM Dagenais GR Diaz R Dyal L. Similar cardiovascular outcomes in patients with diabetes and established or high risk for coronary vascular disease treated with dulaglutide with and without baseline metformin. Eur Heart J. (2021) 42:2565–73. 10.1093/eurheartj/ehaa777 33197271

[56] Rados DV Falcetta MRR Pinto LC Leitao CB Gross JL. All-cause mortality and cardiovascular safety of basal insulin treatment in patients with type 2 diabetes mellitus: a systematic review with meta-analysis and trial sequential analysis. Diabetes Res Clin Pract. (2021) 173:108688. 10.1016/j.diabres.2021.108688 33549676

[57] Mannucci E Targher G Nreu B Pintaudi B Candido R Giaccari A. Effects of insulin on cardiovascular events and all-cause mortality in patients with type 2 diabetes: a meta-analysis of randomized controlled trials. Nutr Metab Cardiovasc Dis. (2022) 32:1353–60. 10.1016/j.numecd.2022.03.007 35422359

[58] Mustafa ST Hofer OJ Harding JE Wall CR Crowther CA. Dietary recommendations for women with gestational diabetes mellitus: a systematic review of clinical practice guidelines. Nutr Rev. (2021) 79:988–1021. 10.1093/nutrit/nuab005 33677540

[59] Hedeager Momsen AM Hotoft D Ortenblad L Friis Lauszus F Krogh RHA Lynggaard V. Diabetes prevention interventions for women after gestational diabetes mellitus: an overview of reviews. Endocrinol Diabetes Metab. (2021) 4:e00230. 10.1002/edm2.230 34277958 PMC8279604

[60] Jowell AR Sarma AA Gulati M Michos ED Vaught AJ Natarajan P. Interventions to mitigate risk of cardiovascular disease after adverse pregnancy outcomes: a review. JAMA Cardiol. (2022) 7:346–55. 10.1001/jamacardio.2021.4391 34705020 PMC8916981

[61] Parikh NI Gonzalez JM Anderson CAM Judd SE Rexrode KM Hlatky MA. Adverse pregnancy outcomes and cardiovascular disease risk: unique opportunities for cardiovascular disease prevention in women: a scientific statement from the American Heart Association. Circulation. (2021) 143:e902–16. 10.1161/CIR.0000000000000961 33779213

